# ASO Author Reflections: Decreased Life Expectancy in Esophageal Cancer Survivors

**DOI:** 10.1245/s10434-022-11563-8

**Published:** 2022-04-04

**Authors:** Ellinor Lundberg, Pernilla Lagergren, Fredrik Mattsson, Jesper Lagergren

**Affiliations:** 1 Upper Gastrointestinal Surgery, Department of Molecular Medicine and Surgery, Karolinska Institute, Karolinska University Hospital, Stockholm, Sweden; 2 Surgical Care Science, Department of Molecular Medicine and Surgery, Karolinska Institute, Karolinska University Hospital, Stockholm, Sweden; 3 Department of Surgery and Cancer, Imperial College London, London, UK; 4 School of Cancer and Pharmaceutical Sciences, King’s College London, London, UK

## Past

Tumor recurrence and related death in esophageal cancer typically occurs soon after esophagectomy (within 1–3 years), and patients who survive > 5 years (30–45% of those who undergo surgery) may be considered cured.^1^ However, it is unknown if there are any differences in survival between esophageal cancer survivors and the background population. The overall survival in esophageal cancer survivors might be worse, e.g. due to risk factors and treatment sequelae, or better, e.g. due to patient selection, lifestyle changes, and symptom awareness.^2^

## Present

The present population-based cohort study showed that the relative survival of the esophageal cancer survivors was initially similar to that of the background population, but decreased with longer follow-up.^3^ The relative survival decreased from 96.1% (95% confidence interval [CI] 94.3–97.9%) postoperative year 6 to 83.5% (95% CI 79.5–87.6%) postoperative year 10. The drop in relative survival was more pronounced in survivors of squamous cell carcinoma of the esophagus than those with adenocarcinoma, and in men than in women. The pattern was similar across age groups and comorbidity scores.^3^

## Future

The results of this first study on the topic need to be confirmed in future studies; the underlying causes for the shorter life expectancy remain to be identified. Such research may lead to recommendations of lifestyle changes (e.g. regarding tobacco smoking and alcohol consumption) and more tailored follow-up of esophageal cancer survivors.
